# Effects of external application of compound Qingbi granules on acute gouty arthritis with dampness-heat syndrome: a randomized controlled trial

**DOI:** 10.1186/s13020-020-00398-8

**Published:** 2020-11-07

**Authors:** Shuang Ren, Fanyan Meng, Yantong Liu, Yun Meng, Ning Tao, Ruoshi Liu, Jie Zhang

**Affiliations:** 1 Department of Traditional Chinese Medicine, The First Hospital of China Medical University, Shenyang, 110001 China; 2 Key Laboratory of Ministry of Education for TCM Viscera-State Theory and Applications, Ministry of Education of China (Province-Ministry Co-Construct), Shenyang, China

**Keywords:** Acute gouty arthritis therapy, External application of traditional chinese medicine, Regulation of inflammatory response, Therapeutic effects

## Abstract

**Background and aim:**

The use of anti-inflammatory and analgesic drugs such as nonsteroidal anti-inflammatory drugs(NSAIDs) for treating acute gout has limitations, such as adverse reactions in the gastrointestinal tract and toxicity in the liver, kidney, and heart. Hence, a new safe and effective treatment approach needs to be explored to reduce the use of anti-inflammatory and analgesic drugs, incidence of adverse reactions, and patients’ burden. This randomized controlled clinical trial aimed to investigate the clinical efficacy and safety of the external application of compound Qingbi granules (CQBG) in treating acute gouty arthritis(AGA), providing evidence for designing a safe, effective, and optimized protocol for AGA comprehensive treatment.

**Methods:**

A total of 90 patients in line with the diagnostic standard of AGA were recruited and randomly divided into control, T1, and T2 groups (30 in each group). All the participators in the three groups all received Western-medicine-basic treatment (low-purine diet, drinking water more than 2000 mL/days, oral loxoprofen, and NAHCO_3_). Besides, the T1 group received an external application of diclofenac diethylamine emulgel, while the T2 group received an external application of CQBG. The participants in the control group received single-use Western-medicine-basic treatment. With a treatment course of 7 days and a follow-up of 7 days, the three groups were compared in terms of primary outcome indicators, including swelling, pain improvement, and change in pain duration and secondary outcome indicators, including serum C-reactive protein (CRP) level, uric acid (UA) level, and change in the thickness of the inflammatory synovium of joints under ultrasound. Meanwhile, the safety of the protocol was evaluated.

**Results:**

The three groups of patients had no apparent differences in age, body mass index, history of gout, complications, and so on before recruitment. A comparison between pretreatment and post-treatment revealed remarkable reductions in the arthralgia visual analog scale score(VAS) and the swelling score in the three groups after the treatment and the improvements in the T2 group were more significant than those in the T1 and control groups (*P* < 0.05). Regarding the onset time of pain improvement and pain duration, the T2 group had more significant efficacy compared with the other two groups (*P* < 0.05). The serum CRP and blood UA levels in the three groups significantly decreased after the treatment, but with no significant intergroup difference. The improvement in the thickness of the inflammatory synovium in joints tested by ultrasound was more significant in the T2 group than in the control group (*P* < 0.05). For safety evaluations, no significant difference in the incidence of adverse events was found.

**Conclusions:**

The external application of CQBG combined with Western-medicine-basic treatment in patients with AGA improved arthralgia and swelling, shortened the period of taking NSAIDs, and reduced the levels of CRP and serum UA. Its therapeutic effect was significantly better than the effect of single-use Western-medicine-basic treatment. The study provided evidence for the clinical application of CQBG combined with Western medicine in treating AGA.

*Trial registration*: ChiCTR, ChiCTR1800018020. Registered 27 August 2018, https://www.chictr.org.cn/showproj.aspx?proj=27138

## Background

Gout is the most common inflammatory arthropathy, which has become a public health concern [1–5]. Acute gouty arthritis (AGA) is clinically characterized by severe arthralgia, redness and swelling of joints, and restricted movement, seriously impacting the quality of life and social functioning of patients due to its easy relapse and difficult cure [1]. The incidence of gout in China continues to increase every year with the change in modern diet structure; it is 8.6% in men [2] and more common in young individuals, gaining increasing attention in the clinic. The priority in the clinical treatment of AGA is to quickly control the acute inflammatory reaction and reduce arthralgia. Colchicine, nonsteroidal anti-inflammatory drugs (NSAIDs) and glucocorticoids are recommended as the first-line treatment of AGA [3, 4]; however, their use may be limited by contraindications commonly reported in patients with gout [5]. For example, colchicine rapidly relieves pain through inhibiting inflammatory reactions. However, its therapeutic dose is similar to the toxic dose, which may cause common gastrointestinal reactions. Besides, it leads to damage in the liver and kidney. NSAIDs may exacerbate renal failure [6, 7], hypertension [6, 8], and cardiovascular disease [6, 8]. Similarly, glucocorticoids may exacerbate diabetes and hyperlipidemia [9], also commonly reported in patients with gout [10, 11]. Hence, a novel effective complementary therapy with less toxic (side) effects while improving arthralgia and swelling during acute attacks and recovering joint function needs to be urgently sought.

TCM has received increasing attention due to its good clinical effects on AGA [12]. The theory of TCM believes that dampness-heat is a typical syndrome of AGA, and dampness likes water and heat likes fire, which frequently initiates the pathogenesis of AGA, causing symptoms such as joint swelling and pain, and limited activity. The TCM formulae of clearing heat and removing dampness, such as Simiao Powder formula [13], Zhuye Shigao decoction [14], and Gout decoction [15], have significant effects in terms of improving inflammatory symptoms in patients with gout and reducing the levels of inflammatory markers [C-reactive protein (CRP), and blood uric acid (UA) level]. Meanwhile, Wang et al. [16] carried out an in vivo experiment to clarify the action mechanisms of Simiao Powder on AGA. The study reported that the mechanisms were relevant to significantly suppress the expression of inflammatory cytokines IL-6, TNF-α, and IL-1β in the joints of rats with AGA. However, few studies were performed on the external application of TCM in treating AGA. Clinically, the external application of TCM is an important treatment mode with a long history and clear therapeutic efficacy recorded in Chinese medicine classics. It is suitable for patients not comfortable with oral administration and has the advantage of a lower absorbed dose of the drug percutaneously. Recent reports indicated that the effect mechanism of the external application of TCM might correlate with improving local microcirculation, and promoting inflammatory absorption [17].

CQBG were developed based on the syndrome differentiation and treatment in TCM combined with the advantages of external treatment and applied to treat the dampness-heat syndrome in arthritis; it had *Cortex Phellodendri* and *Herba tuberculate speranskia* as the main components. Previous clinical observations indicated that the external application of CQBG quickly alleviated symptoms of dampness-heat syndrome in patients with osteoarthritis. CQBG have been applied in arthritis for many years, and current pharmacological researches have supplied the evidence about the effect of CQBG on AGA [18–20]. However, trials on its efficacy and safety are lacking. Therefore, a randomized, controlled, and follow-up research protocol with a desirable methodology was adopted in this study to assess the efficacy and safety of the external application of CQBG combined with Western-medicine-basic treatment in AGA.

## Materials and methods

### Research design

This trial was designed as an open-label, randomized, controlled, and parallel-group study that focused on the therapeutic efficacy and safety of CQBG combined with Western-medicine-basic treatment in treating AGA. It was conducted from September 2018 to December 2019 in the Department of TCM, the First Affiliated Hospital of China Medical University, which is the NO.1 comprehensive hospital in China's northeast region with a wide range of patients. The study was approved by the ethics committee of the First Affiliated Hospital of China Medical University and registered in the Chinese Clinical Trial Registry. All patients signed the informed consent form.

### Diagnostic criteria

#### Diagnostic criteria of AGA

For the diagnosis of primary gout, one can refer to the gout classification criteria in the 2015 American College of Rheumatology (ACR)/European League Against Rheumatism(ULAR) [21].

Acute attack stage of gout: Signs may not occur before the attack. Typical patients with the acute attack are often awakened by arthralgia, which worsens progressively and reaches the peak about 12 h later, with unbearable bursting pain, cutting pain, and gnawing pain. The involved joints present swelling, burning, tight skin, obvious tenderness, and restricted motion. The attack can voluntarily alleviate and return to normal in a few days (up to 2 weeks). The first attack often involves a single joint, with more than 50% in the first metatarsophalangeal joint and 90% in the later course. Besides, joints such as dorsum pedis, heel, ankle, and knees can be involved. Symptoms such as fever, chill, headache, palpitation, and nausea can occur in some patients with an increased number of white blood cells, CRP level, and erythrocyte sedimentation rate, showing urate crystals in ultrasound.

Hyperuricemia: a test of blood UA levels twice on different days: sUA > 420 mmol/L.

#### Differentiated criteria of the dampness-heat syndrome

The differentiation of the dampness-heat syndrome was confirmed by two TCM professors based on clinical symptoms and signs, as well as pathogen and pathological mechanism, including swelling and heat pain in local single joints or multiple joints, accompanied by fever, fear of cold, thirst, anxiety, headache, sweating, less and yellow urination, red tongue, yellow or greasy tongue coating, and stringy and rapid pulse, which complied with *Criteria of Syndrome Differentiation and Therapeutic Effect of Zhuoyubi* (*gouty arthritis*) *in TCM* published by the State Administration of Traditional Chinese Medicine (2017 edition) and *Criteria of Diagnosis and Therapeutic Effect of Internal Diseases and Syndromes in Traditional Chinese Medicine* published by the State Administration of Traditional Chinese Medicine of the People’s Republic of China (ZY/T001.1–94).

#### Recruitment criteria

The inclusion criteria were as follows:

(1) conforming to the diagnostic criteria of AGA and hyperuricemia; (2) diagnosis of dampness-heat syndrome; (3) age 18–70 years and any sex; (4) AGA attacked ≥ 1 in the previous year; (5) alleviation period in previous AGA attacks, ≤ 14 days; (6) main observed regions including first metatarsophalangeal joint, dorsum pedis, ankle joint, knee joint, and so forth, and only the most severe joint (target joint) observed and recorded for each participant, with no change during the observation; (7) VAS score (evaluation of pain scoring criteria) in the target joint, ≥ 3; (8) < 72 h between the last treatment and the attack; and (9) patients who voluntarily participated and signed the written informed consent form.

#### Exclusion criteria

The exclusion criteria were as follows:

1. Secondary gout or arthropathy caused by other diseases (e.g., rheumatic arthritis, pyogenic arthritis, traumatic arthritis, senile osteoarthritis, pseudogout, chemotherapy, radiotherapy, chronic lead poisoning, and acute obstructive nephropathy)
2. Chronic intermittent gout or chronic tophaceous gout
3. More than four joints involved in the AGA attack
4. Patients taking drugs that affected the metabolism of blood UA, for example hydrochlorothiazide, furosemide, low-dose aspirin, and drugs that contained the aforementioned components, such as compound reserpine and hydrochlorothiazide; or patients who stopped taking glucocorticoids less than 1 month before enrollment; or patients using NSAIDs, or other analgesic drugs, or external ointment 24 h before the baseline assessment
5. Severe malformation because of gouty arthropathy or disability resulting from stiffness
6. Pregnancy or lactation
7. Allergic constitution or a history of allergy
8. Serum creatinine (Scr) exceeding the upper limit of the reference value
9. Liver function, Alanine aminotransferase(AST) and Aspartate aminotransferase(ALT) levels 1.5 times higher than the normal upper limit
10. Clinically significant arrhythmia
11. History of alcohol or drug abuse
12. Severe cerebrovascular, renal, liver, or hematopoietic comorbidities, cancer, or mental disorders
13. Participated in other clinical trials in the last 3 months
14. Referring to the judgment by investigators: some other diseases or situations leading to a lower possibility of recruitment or complicate the enrollment, such as missing visits due to frequent changes in the workplace.

### Randomization, blinding and intervention

#### Randomization

Patients with AGA were randomly divided into three groups. Following the distribution sequence, random numbers were created using SAS9.2 edition (Straits Leading Pharmaceutical R&D Co. Ltd., Heping District of Shenyang) by an independent statistician from the CMU1h Clinical Trials (GCP) center. Every random number was put into a serially numbered opaque envelope and screened by clinical coordinators. After screening, the clinical researchers provided patients with treatment according to the randomized serial number.Every eligible patient was given a specific treatment number, which was a fixed number for the whole trial used as the basis of drug allocation.

#### Blinding

The blinding method was not suitable for both patients and evaluators due to the obvious difference in pharmaceutical types between CQBG used in the treatment group 2 and diclofenac diethylamine emulgel used in the treatment group 1. However, the statisticians were blinded to the study design.

#### Intervention

A total of 90 patients in line with the diagnostic standard of AGA were recruited and divided randomly into control, T1, and T2 groups (30 in each group). The participants in the three groups all received Western-medicine-basic treatment, including low-purine diet, drinking water more than 2000 mL/days, three times loxoprofen (60 mg each time) and NAHCO_3_ (1 g each time) per day orally. Besides, the T1 group received an external application of diclofenac diethylamine emulgel which is anti-inflammatory and analgesic drugs commonly used in clinic and produced by Novartis Pharma (Beijing) Stein AG. The T2 group received an external application of CQBG. CQBG were prepared adhering to the national production standard by Jiangyin Tianjiang Pharmaceutical Co. Ltd., Jiangsu province. They have Cortex Phellodendri and Herba tuberculate speranskia as the main components. The components and quality control mapping of CQBG are shown in Additional file 1.

External medicine usage: Before application, each pack of CQBG (30 g) was dissolved in 80 mL of water, and mixed well to form a paste. Apply CQBG or diclofenac diethylamine emulsifier evenly on the affected area. The dosage of CQBG or diclofenac diethylamine emulsifier was defined (1 cm outside the painful area; local application thickness 1–2 cm [22], 3 times a day). The participants in the control group received single-use Western-medicine-basic treatment. The total treatment course lasted 7 days and the patients in the three groups were followed up for 7 days.

### Ethics permission and registration

This study was performed following the standard of the International Coordinating Committee on Global Partnerships and the revised edition of the Declaration of Helsinki. It was registered in ChiCTR (ChiCTR1800018020). Every participant endorsed the informed consent voluntarily.

### Observation indicators

#### Primary clinical outcome indicators

1. Change in the VAS score in the target joint: 0 for no pain and 10 for unbearable pain [23]. The VAS score was evaluated three times a day with consistency among groups in terms of patients’ feeling of pain, and its mean value was taken as the VAS score of the day.
2. Onse time of pain improvement in the target joint: VAS < 3 was defined as pain improvement [24].
3. Change in pain duration in the target joint: The duration of target joint pain was obtained from a daily pain recording card, and the change in pain duration was defined as the pain duration on the testing day minus the pain duration on the previous day. To reduce deviation, the patients were stratified into groups 0–24 h, 24–48 h, and 48–72 h according to the disease course of acute gout. The change in pain duration on days 1–4, 7, 10, and 14 was observed.
4. Swelling score: 1 for no arthrocele, 2 for palpable arthrocele, 3 for macroscopic arthrocele, and 4 for swelling exceeding the joint edge [23, 25]. The swelling change in all groups on days 0, 7, and 14 was observed.

#### Secondary outcome indicators

UA, CRP, and ultrasound examinations of the thickness of the inflammatory synovium of joints were evaluated once before and after the treatment.

### Safety evaluation

Examinations including physical examination, blood routine, urine routine, and hepatorenal function as well as records of all adverse events, were assessed and analyzed with drug dependency.

### Statistical analysis

Data were analyzed with SPSS 23.0 software, while measurement data were presented with ${\bar{\text{x}}} \pm {\text{s}}$. Per-protocol analysis (PPS) was used to analyze data in the present study. Some baseline characteristics were assessed by one-way analysis of variance (ANOVA). Mauchly’s test of sphericity should be used to judge whether there were relations among the repeated measured data. If any (*P* < 0.05), repeated measures and multivariate analysis of variance of the general linear model should be taken. When Mauchly’s test of sphericity is *P* > 0.05, univariate ANOVA can be used. Bonferroni, LSD and S-N-K tests were chosen for multiple comparison and multiple correction. While qualitative variables were compared using the Chi-squared test (χ^2^), or the Fisher’s exact test in the case of small sample size. A *P* value < 0.05 indicated a statistically significant difference.

## Results

### General data

**Fig. 1:**
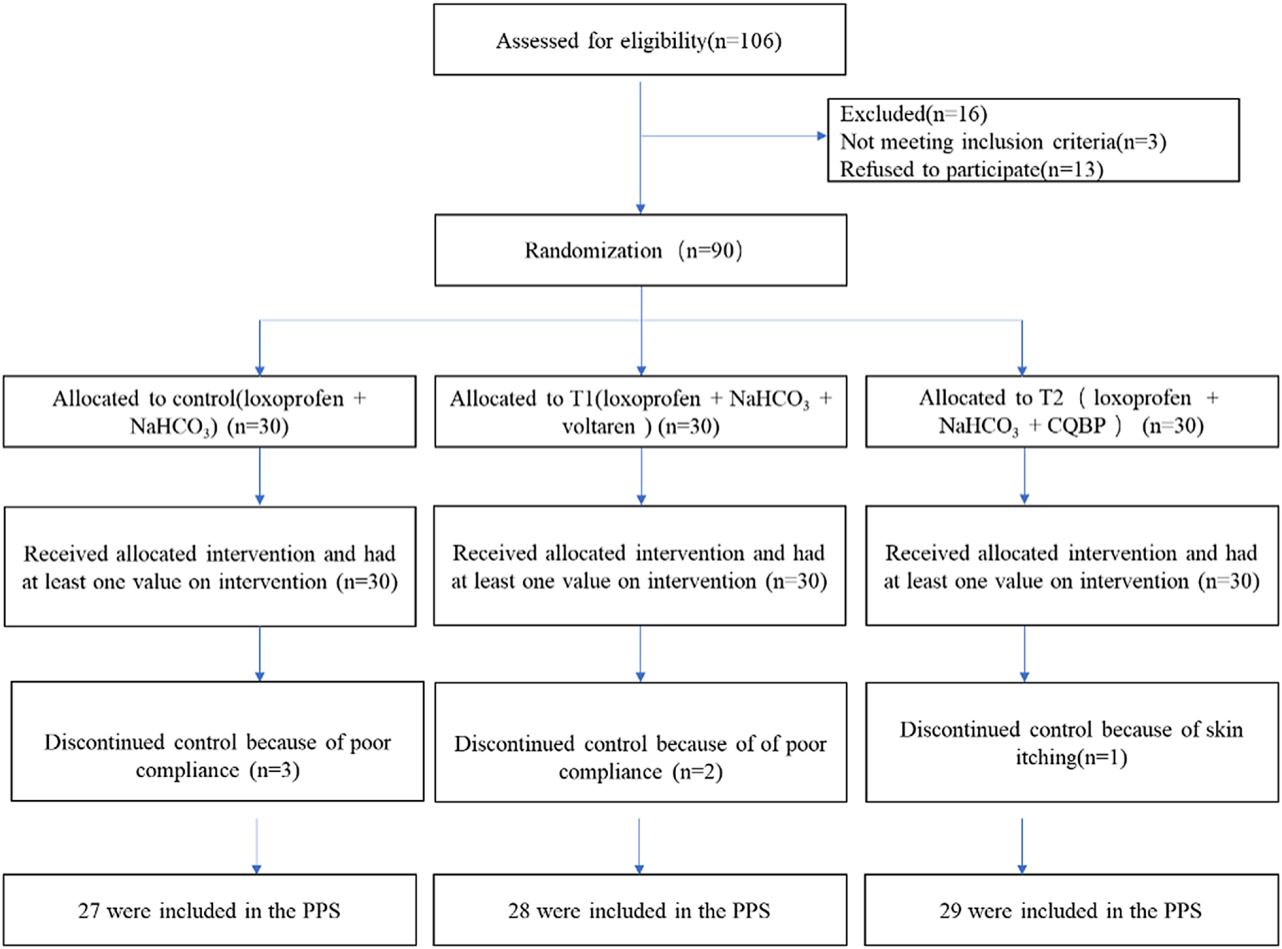
Patient flowchart

A total of 90 patients were recruited and distributed into the control, T1, and T2 groups in a ratio of 1:1:1. Three patients from the control group and two from the T1 group failed to complete the study due to their bad compliance, while one from the T2 group could not complete the study because of pruritus. Table 1 shows the baseline characteristics of patients, and Fig. 1 shows the recruitment procedure. No differences were found in the demographic data of all groups (*P* > 0.05).

**Table 1:** Demographic and baseline characteristics.

Variable	Control group n = 27	Treatment group1 n = 28	Treatment group2 n = 29
Age (years, mean ± SD)	45.00 ± 3.17	48.00 ± 7.05	46.00 ± 7.49
Men, n (%)	19 (70.37%)	20 (71.43%)	19 (65.52%)
History of gout (years, median (range))	3.00 (0–9)	3.00 (0–10)	3.00 (0–11)
BMI (kg/m^2^, mean ± SD)	32.00 ± 5.07	31.07 ± 4.00	32.05 ± 5.99
Uric acid (pre-treatment) (μmol/L, mean ± SD)	480.04 ± 98.04	470.14 ± 90.12	479.97 ± 97.96
Onset time, n (%)			
≤ 24 h	3 (11.11%)	4 (14.29%)	5 (17.24%)
24–48 h	9 (33.33%)	8 (28.57%)	8 (27.59%)
48–72 h	15 (55.56%)	16 (57.14%)	16 (55.17%)
Index joint, n (%)			
Metatasophalangeal joint 1	4 (14.81%)	3 (10.71%)	4 (13.79%)
Other foot joints,	5 (18.52%)	5 (17.86%)	9 (31.03%)
Ankle	5 (18.52%)	6 (21.43%)	7 (24.14%)
Knee	6 (22.22%)	9 (32.14%)	7 (24.14%)
Wrist	1 (3.70%)	1 (3.57%)	0
Hand	2 (7.41%)	3 (10.71%)	2 (6.90%)
Elbow	1 (3.70%)	0	0
Multiple joints	3 (11.11%)	1 (3.57%)	0
Joint swelling, n (%)			
No swelling	0	2 (7.14%)	0
Palpable	6 (22.22%)	8 (28.57%)	8 (27.59%)
Visible	6 (22.22%)	8 (28.57%)	10 (34.48%)
Bulging beyond joint margins	15 (55.56%)	10 (35.71%)	11 (37.93%)
Activity, n (%)			
No restricted	0	0	0
Moderate restricted	5 (18.52%)	7 (25.00%)	9 (31.03%)
Significantly restricted	9 (33.33%)	11 (39.29%)	10 (34.48%)
Unbearable, cannot take care of themselves	13 (48.14%)	10 (35.71%)	10 (34.48%)
Smoke use, n (%)	5 (18.52%)	7 (25.00%)	6 (20.69%)
Drink, n (%)	10 (37.04%)	11 (39.29%)	11 (37.93%)

^a^No significant differences were found in the demographic data of all groups (*P* all  > 0.05)

### Main outcome indicators

#### Comparison of the VAS score of the target joint on days 1, 2, 3, 4, 7, and 14 in the three groups

**Fig. 2:**
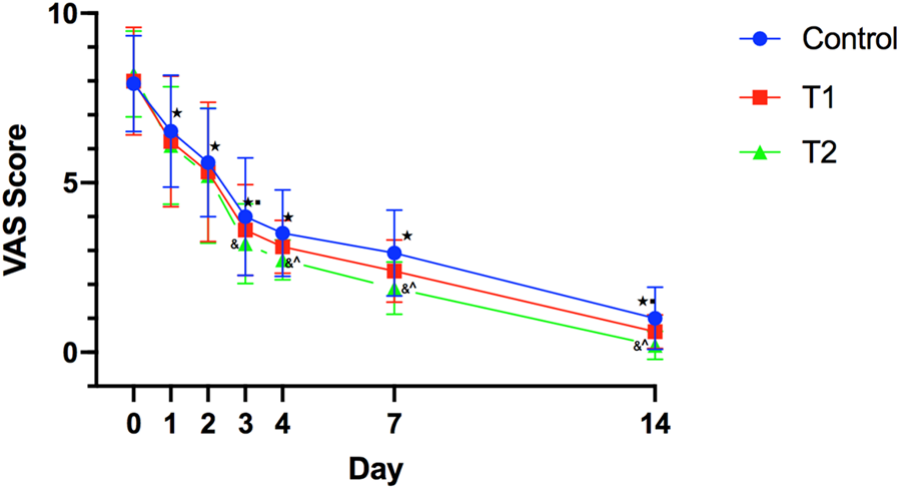
Comparison of the mean changes of patients VAS score in the three groups. Statistical significant difference between before and after treatment in each group ^★^*P* < 0.05; Statistical significant difference between treatment group 2 and control group ^&^P < 0.05; Statistical significant difference between treatment group 2 and treatment group 1 ^P < 0.05; Compared with the previous time-point of this group ^■^ *P* < 0.05

After treatment, VAS scores of the target joint in the three groups decreased on days 1, 3, 5, and 7; some of the changes were statistically significant (*P* < 0.05). The VAS score on day 3 was significantly lower in the T2 group than in the control group (*P* < 0.05). The VAS scores on days 4 and 7 in treatment and day 14 in observation were significantly lower in the T2 group than in the T1 and control groups, as shown in Fig. 2.

#### Comparison of change in pain duration of the target joint in the three groups

A gradual decline in the pain duration of the target joint in the three groups was observed after the treatment. From day 2, the reduction in pain duration was significantly better in the T2 group than in the control group (*P* < 0.05); on day 4, the reduction in pain duration was much better in the T2 group than in the T1 group (*P* < 0.05), as shown in Table 2. Significant differences were observed on days 1, 4, 7 and 14 among different stratified blocks of pain duration(*P* < 0.05).

**Table 2:** Comparison of change in pain duration change in the target joint in the three groups

	Pain duration	Group	After treatment					
			1 day	2 day	3 day	4 day	7 day	14 day
Changes in pain duration	< 24 h	Control group	1.00 ± 0.33	2.20 ± 0.55	4.50 ± 0.33	4.00 ± 0.66	1.30 ± 0.22	0.10 ± 0.22
		Treatment group1	1.10 ± 0.18	2.50 ± 0.50	4.60 ± 0.50	4.20 ± 0.50	1.40 ± 0.38	0.20 ± 0.25
		Treatment group2	1.30 ± 0.36	2.60 ± 0.32^*^	4.80 ± 0.32^*^	4.50 ± 0.36^*#^	1.70 ± 0.20^*#^	0.10 ± 0.32^*#^
	24–48 h	Control group	1.20 ± 0.29	2.40 ± 0.43	4.00 ± 0.67	3.50 ± 0.72	2.20 ± 0.29	0.20 ± 0.26
		Treatment group1	1.30 ± 0.39	2.70 ± 0.48	4.20 ± 0.48	3.60 ± 0.47	2.30 ± 0.39	0.20 ± 0.28
		Treatment group2	1.50 ± 0.38	3.00 ± 0.25^*^	4.60 ± 0.57^*^	3.80 ± 0.31^*#^	2.50 ± 0.50^*#^	0.20 ± 0.11^*#^
	48-72 h	Control group	1.70 ± 0.52	2.30 ± 0.68	3.00 ± 1.07	3.10 ± 0.48	2.50 ± 0.47	0.20 ± 0.24
		Treatment group1	1.40 ± 0.36	3.00 ± 0.75	4.60 ± 1.3	3.40 ± 0.47	2.60 ± 0.38	0.30 ± 0.23
		Treatment group2	1.60 ± 0.30	3.50 ± 0.75^*^	3.50 ± 1.53^*^	3.50 ± 0.85^*#^	2.90 ± 0.42^*#^	0.20 ± 0.06^*#^

^*^P < 0.05, *vs* control group at the same time, ^#^*P* < 0.05, *vs* T1 group at the same time, there was a statistically significant difference (*P* < 0.05)

#### Onset-Time comparison of pain improvement of the target joint in the three groups

**Fig. 3:**
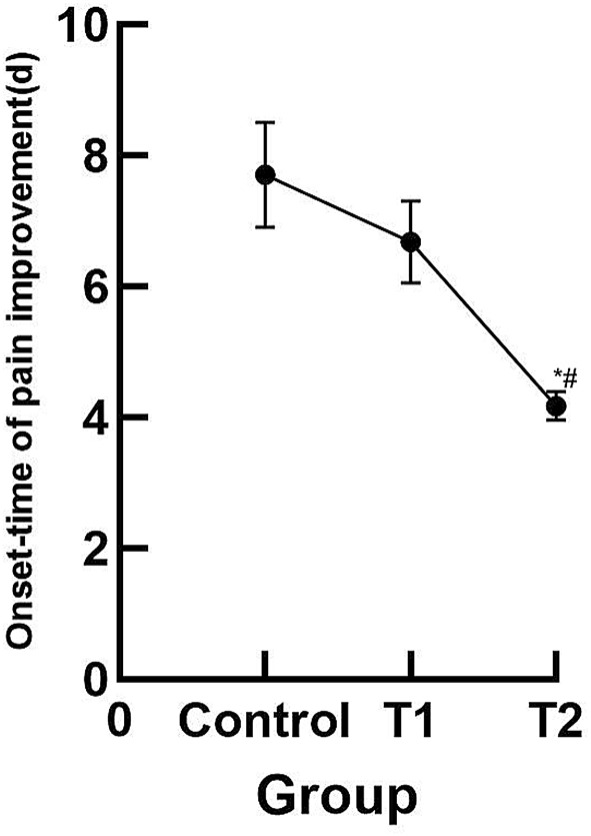
Comparison of onset-time of pain improvement of the target joint in three groups. ^*^P < 0.05, *vs* control group, ^#^*P* < 0.05, *vs* T1 group, there was a statistically significant difference (*P* < 0.05)

Pain improvement of the target joint in the T2 group occurred earlier than that in the control and T1 group with a statistically significant difference (*P* < 0.05), as shown in Fig. 3.

#### Comparison of the swelling score of the target joint in the three groups

**Fig. 4:**
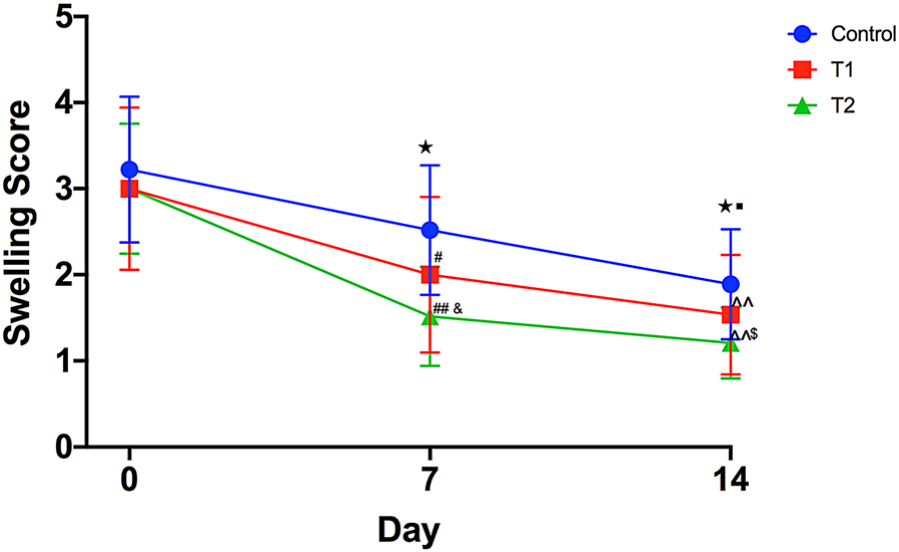
Comparison of swelling score in three groups on 7,14 day. *vs* Day 0, ^★^*P* < 0.05; *vs* Day 7, ^■^*P* < 0.05; On Day 7, *vs* Control, ^##^*P* < 0.01, ^#^*P* < 0.05; *vs* T1, ^&^*P* < 0.05; On Day 14, *vs* Control, ^^*P* < 0.01; *vs* T1, ^$^*P* < 0.05

The analysis of repeated measurement and comparison between pretreatment and post-treatment showed that the swelling scores on days 7 and 14 in the three groups decreased remarkably (*P* < 0.05). The swelling score on day 7 was significantly lower in the T2 group than in control (*P* < 0.01) and T1 groups (*P* < 0.05) while the score in the T1 group was significantly lower than that in the control group (*P* < 0.05). On observation day 14, the swelling score was significantly lower in the T2 group than in the control (*P* < 0.01) and T1 groups (*P* < 0.05), and significantly lower in the T1 group than in the control group (*P* < 0.01), as shown in Fig. 4.

### Comparison of change in CRP level, UA level, and thickness of the synovium of target joints before and after the treatment in the three groups

**Fig. 5:**
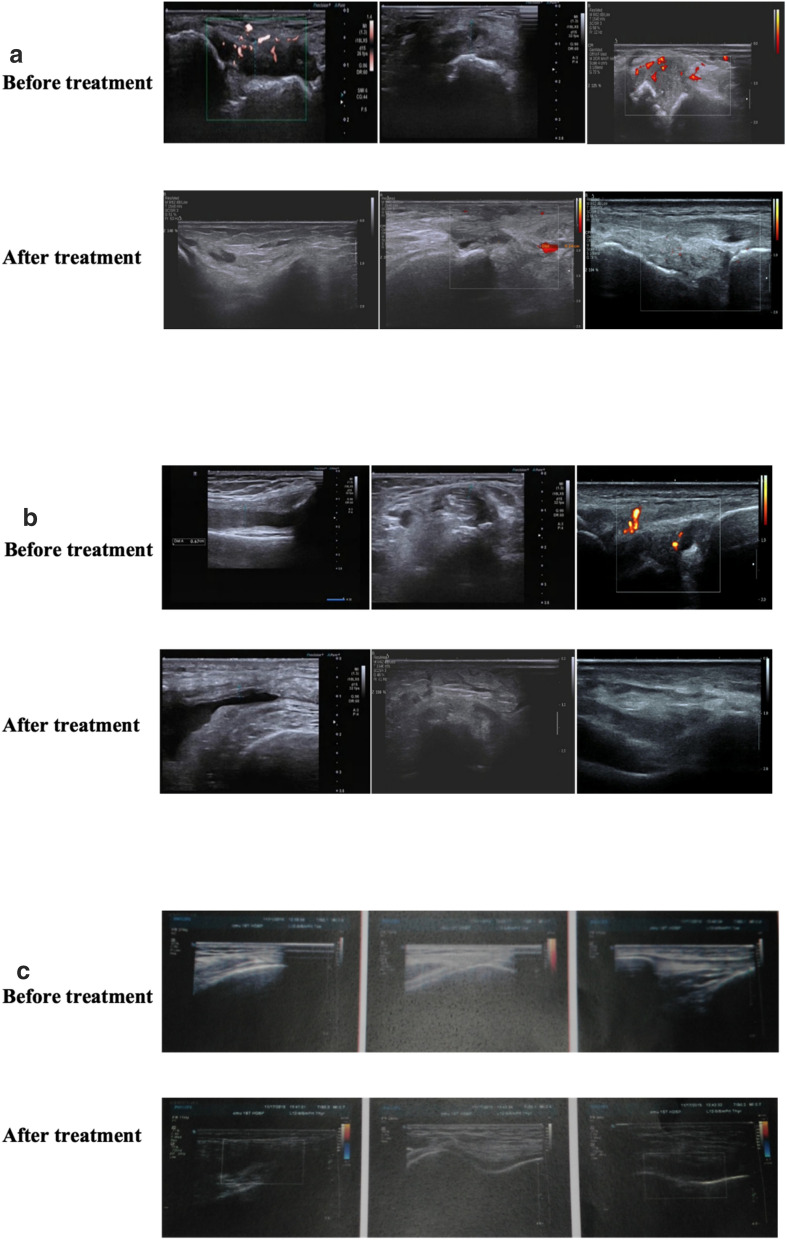
The measurement of synovial thickness of joint under color ultrasound in Control group (**a**), T1 group (**b**), T2 group (**c**)

Compared with pretreatment, CRP and blood UA levels in the control and treatment groups decreased significantly after the treatment (*P* < 0.05). Still, no statistically significant difference was observed among the three groups, as shown in Table 3. In terms of change in the thickness of the synovium of target joints after the treatment, a significant improvement was seen in the T2 group (*P* < 0.05), which was better than that in the control group (basic treatment group), as shown in Table 4 and Fig. 5.

**Table 3:** Comparison of the average changes of CRP, Urine urate in the three groups

	Group	Before treatment	7 days	14 days
CRP (mg/L)	Control group	45.07 ± 22.08	13.96 ± 3.97^*^	7.00 ± 2.96^*#^
	Treatment group1	46.04 ± 24.97	11.04 ± 4.05^*^	6.07 ± 3.00^*#^
	Treatment group2	47.03 ± 23.07	12.97 ± 5.00^*^	5.06 ± 2.99^*#^
Serum uric acid (μmol/L)	Control group	480.04 ± 98.04	449.96 ± 90.04	402.00 ± 60.00^*#^
	Treatment group1	470.14 ± 90.12	440.04 ± 86.98	390.00 ± 55.14^*#^
	Treatment group 2	479.97 ± 97.96	440.00 ± 74.97	390.00 ± 60.05^*#^

^*^P < 0.05, *vs* before treatment at the same group, ^#^*P* < 0.05, *vs* 7 days at the same group,

there was no statistically significant difference among the three groups (*P* > 0.05)

**Table 4:** Comparison of the average improvements of synovial thickness before and after the treatment in the three groups

	Group	Changes in the patient's synovial thickness (cm)
Synovial thickness	Control group	0.373 ± 0.05
	Treatment group1	0.394 ± 0.06
	Treatment group 2	0.412 ± 0.07^*^

^*^P < 0.05, *vs* control group

### Safety evaluation

No severe adverse event was observed in the three groups. The cumulative incidence of adverse reactions in the T1, T2, and control groups was 10.7%, 6.9%, and 7.4%, respectively, showing no statistically significant difference in the three groups, as shown in Table 5.

**Table 5:** Adverse reactions

	CONTROL group	Treatment group1	Treatment group2
Total adverse effects	2/27	3/28	2/29
Gastric or abdominal pain	2 (7.4%)	2 (7.1%)	1 (3.4%)
Edema	0	1 (3.6% ()	0
Skin Itch	0	0	1 (3.4%)

Additionally, the incidence of gastric or abdominal pain was higher in the control group than in the T2 group. Therefore, it was hypothesized that CQBG treatment contributed to the attenuation of gastric or abdominal pain against the side effect of loxoprofen on the gastrointestinal tract.

## Discussion

The incidence of gout continues to increase every year, and an acute attack is often triggered by multiple factors in its chronic course. NSAIDs, systemic corticosteroids, and oral colchicine are recommended by the 2012 ACR guide for treating AGA [26]; however, the side effects of these drugs limit their use in the clinic. Recent studies on AGA treatment with TCM have shown unique advantages and fewer side effects [12]. In this study, the T2 group had a significant reduction in the VAS score of joint pain, arthrocele, and pain duration compared with the control and T1 groups (*P* < 0.05), indicating that the external application of CQBG, combined with the Western-medicine-basic treatment, quickly alleviated clinical symptoms such as pain and swelling, shorten disease course, and reduced patients’ burden.

The dampness-heat syndrome is a common syndrome of AGA in the clinic. In the theory of TCM, a deficiency of spleen qi and an imbalance of metabolism of water and food can induce the accumulation of damp. Additionally, eating too much greasy food with stronger flavor can cause the accumulation of damp, leading to heat accumulation in the skin and joints and resulting in a gout attack. Hence, the external application of TCM for gout focused mainly on clearing heat and expelling dampness, which was demonstrated in some studies with apparent efficacy against AGA, namely, good therapeutic effect in controlling symptoms in acute gout and reducing recurrence rate. For instance, oral Jiawei Simiao Powder and external application of Sihuang water–honeyed pill had significant effects in decreasing the blood UA level and improving the joint function of patients with AGA [27].

In this study, CQBG were found to be effective in improving the inflammation and pain of patients with AGA. Its mechanism might be related to the decline in inflammatory cytokine release caused by effective components of this compound drug. CQBG is formulated with Cortex Phellodendri and Herba tuberculate speranskia. Cortex Phellodendri with cold nature and originally documented in the Holy Husbandman's Classic on Roots and Herbs (finished in 1616 AD); it was always used against pyogenic infections with active elements of tetrandrine and berberine that exerted anti-inflammatory and immunoregulatory effects by reducing the expression of inflammatory cytokines and increasing the expression of anti-inflammatory cytokines [28, 29]. Clinically, Cortex Phellodendri can be used in the treatment of arthritis, gout, and so forth, through lowering UA and creatinine levels in model rats with hyperuricemia and inhibiting arthrocele in model rats with acute gout arthritis [30]. Herba tuberculate speranskia, which is pungent and warm in nature, was originally documented in Jiuhuang Beneao (finished in 1525 AD) with the efficacy of expelling damp and swelling and relieving pain. The anti-inflammatory effect of this Chinese traditional medicinal crop and its components and the inhibition of platelet aggregation were shown in pharmacological studies. Moreover, this drug reduced the swelling of the paw in model rats with arthritis; inhibited the proliferation and transfer of synovial cells and release of inflammatory cytokines; decreased the protein expression of inflammasome NLRP3, caspase-1, and IL-1β; inhibited inflammatory cell infiltration and angiogenesis; promoted apoptosis; reduced serum inflammatory factors IL-1β and TNF-α levels; and significantly inhibited the inflammatory reaction. Clinically, Herba tuberculate speranskia is used mostly for external application in rheumatic arthralgia, bone and muscle contracture, and pyogenic infections [20, 31–35]. In summary, the external application of this compound had antibacterial, anti-inflammatory, analgesic, and anticoagulatory effects against AGA. Among these, the anti-inflammatory function helped reduce the local swelling of joints and prevent local infection. The analgesic effect relieved anxiety produced by pain, and the anti-coagulatory effect helped in the remission of local swelling and pain besides thrombogenesis prevention. Moreover, drugs were efficiently absorbed percutaneously, achieving an obvious clinical effect in a short time.

Any effective anti-inflammatory drugs for external use must enter the dermis and subcutaneous tissues through the skin, and then reach the inflammatory tissues through local blood supply to play an analgesic role by inhibition of the synthesis of cyclooxygenase (COX) and prostaglandin. In our study, CQBG was found to be effective in improving the inflammatory symptoms of patients with AGA including pain, and swelling evaluated by different indicators and methods, but no statistically significant difference in CRP and UA levels was observed among the three groups, suggesting that it did not affect CRP and UA level.

The priority in the clinical treatment of AGA is to quickly control the acute inflammatory reaction. NSAIDs are one of the first-line treatments, and medicines lowering UA level generally are not recommended in the acute phase. NSAIDs including diclofenac diethylamine emulgel, had good analgesic and anti-inflammatory effects mainly by inhibiting cyclooxygenase (COX) to reduce the conversion of arachidonic acid into PGE2 (prostaglandin E2) and other inflammatory mediators. CRP is a nonspecific indicator reflecting inflammatory activity. The meta-analysis of randomized controlled trials showed that NSAIDs, the first line medicine for the AGA, had no direct effect on the CRP level in rheumatoid arthritis and ankylosing spondylitis [36, 37] which both are inflammatory arthritis. Our previous research on the action mechanisms of CQBP based on network pharmacology suggested that prostaglandinendoperoxide synthase2 (PTGS2), phosphatidylinositol 3-kinase (PIK3), and mitogen-activated protein kinase (MAPK) were the important treatment targets of this granule ( scoring top 3 calculated using Cytoscape 3.7.1 software) [38]. PTGS is a rate limiting enzyme in the initial step of prostaglandin (PG) synthesis, and PTGS2 is a promoter of the development of malignant tumors and inflammation. In the case of tissue injury or inflammation, PTGS2 can be rapidly expressed under the stimulation of a series of pro-inflammatory factors, such as interleukin-1 (IL-1), and catalyze the synthesis of PG which participates in the regulation of the NF-κB inflammation pathway [39, 40]. PI3K signal transduction is involved in the abnormal cell proliferation of immune cells and synovial fibroblasts [41]. MAPK induces fibroblast-like synoviocytes to produce a large amount of collagen, PGE2, MMPs and a variety of inflammatory cytokines by the phosphorylation of Jun N-terminal Kinase(JNK), p38 and extracellular regulated protein kinases(ERK), and finally aggravating the inflammatory reaction [42]. Therefore, the aforementioned pathological process may be the effect mechanisms of CQBG; however, further clinical and experimental studies are needed for confirming the conclusions.

UA exists in vivo as ionic UA salts, and urate crystals start accumulating in tissues when the serum UA level exceeds the normal threshold. A continuous accumulation of urate crystals in joints triggers an acute inflammatory reaction with severe arthralgia, swelling, burning, redness, and difficulty in the movement of involved joints. Hence, the UA level should be controlled below the standard level to avoid an acute attack during the period of gout intermission. However, medicines lowering UA levels generally are not recommended as the first-line treatment in the acute phase [4]. Although, experimental results indicated that the extract of Cortex phellodendri (one of the components of CQBG) significantly inhibited xanthione oxidase (XOD) activity in the liver, downregulated XOD mRNA and protein expression, and significantly reduced the expression level of mURAT1 mRNA and protein in the kidney, thus having dual effects including the inhibition of the production and the reabsorption of UA in the kidney in mice with hyperuricemia [43]. The present study showed that CQBG treatment could lower the blood UA level without any significant difference compared with conventional treatment (control). This might be because of the low content of components that could reduce UA levels in this granule, and the effect was not remarkable.

Ultrasound has the characteristics of easy operation, noninvasiveness, flexibility, and high sensitivity and hence provides vivid and visualized monitoring and assessment for patients with gout [44]. Therefore, it is widely applied in the diagnosis and evaluation of gouty arthritis. Synovium thickening was a typical manifestation of gout in the acute stage. In this study, a significant decline in the thickness of the synovium of joints was observed in the CQBG treatment group compared with the control group. In this study, the detection rate of double-track sign under ultrasound is not high. The reason may be that the patients in this study were in patients in a serious condition, most of them also had apparent gout stones and bone destruction. Hence, the sound attenuation affected the detection rate. At the same time, the double-track sign should be differentiated from calcium pyrophosphate deposition in the joint (also known as pseudogout).

The baseline regarding BMI, history of gout, and UA level was close among the three groups due to the lifestyle and dietary habits of the population in Northeast China, high incidence of obesity in patients with gout, and small sample size. Randomized, multi-center, controlled clinical trials with a large sample size are needed to further confirm the efficacy of CQBG against AGA.

## Conclusions

In this study, the external application of CQBG combined with Western-medicine-basic treatment was used for treating AGA with dampness-heat syndrome, showing good and safe clinical effects in terms of quickly alleviating pain and main clinical symptoms as well as inhibiting the inflammatory reaction. Furthermore, it reduced the use of NSAIDs, and patients’ burden, and improved the quality of life of patients.

## Supplementary information

**Additional file 1: Table S1.** The compositions of CQBP.** Fig. S1. **The fingerprints of CQBP. Peak number and identity, 1: phellodendrine; 2: magnoflorine; 3:jatrorrhizine; 4: tetrandrine; 5: columbamine; 6: phenanthrene herb and alkaloid; 7: berberine; 8: ferulic acid.

## Data Availability

The datasets used in the present study are available from the corresponding author on reasonable request.

## Abbreviations

AGA: Acute gouty arthritis
ASC: Apoptosis-associated speck-like protein containing a CARD
BMI: Body mass index
CQBG: Compound Qingbi granules
CRP: C-reactive protein
ERK: Extracellular regulated protein kinases
GCP: Guidelines for good clinical practice
IL-6: Interleukin-6
JNK: Jun N-terminal Kinase
MAPK: Mitogen-activated protein kinase
MMP: Matrix metalloproteinase
mURAT1: Mouse urate anion transporter 1
NSAIDs: Nonsteroidal anti-inflammatory drugs
NF: Nuclear factor
NLRP3: NOD-like receptor family, pyrin domain containing 3
PGE2: Prostaglandin E2
PTGS2: Prostaglandinendoperoxide synthase2
PIK3: Phosphatidylinositol 3-kinase
TLR: Toll-like receptor
TNF-α: Tumor necrosis factor-alpha
UA: Uric acid
VAS: Visual analog scale/score
XOD: Xanthione oxidase
