# Supplementary material for: Effects of external application of compound Qingbi granules on acute gouty arthritis with dampness-heat syndrome: a randomized controlled trial
Source: Chin Med. 2020 Nov 7;15:117. doi: 10.1186/s13020-020-00398-8 (PMC7648992; doi:10.1186/s13020-020-00398-8)
Supplement: Supplementary file 1 — Additional file 1: Table S1. The compositions of CQBP. Fig. S1. The fingerprints of CQBP. Peak number and identity, 1: phellodendrine; 2: magnoflorine; 3:jatrorrhizine; 4: tetrandrine; 5: columbamine; 6: phenanthrene herb and alkaloid; 7: berberine; 8: ferulic acid. [file 13020_2020_398_MOESM1_ESM.docx]

**Additional file 1:**

**Additional file 1: Table S1 The compositions of CQBP**

| **NO.** | **Herbal drug** | **Local name** | **Collection place** | **Batch number** |
| --- | --- | --- | --- | --- |
| 1 | Cortex phellodendri | Huangbai | Sichuan | 19072081 |
| 2 | Herba tuberculate speranskia | Tougucao | Jilin | 19072871 |


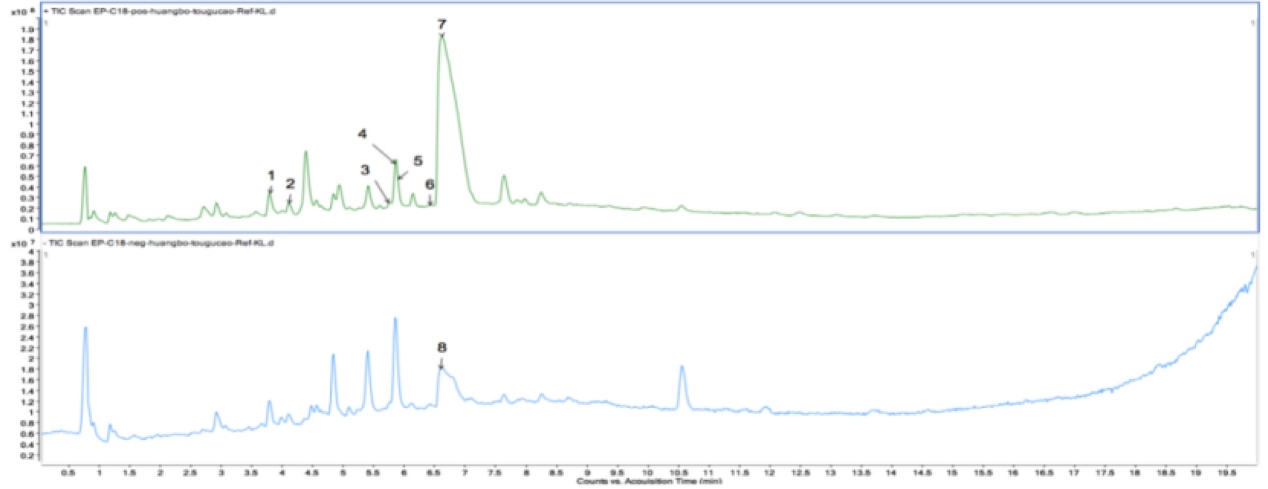


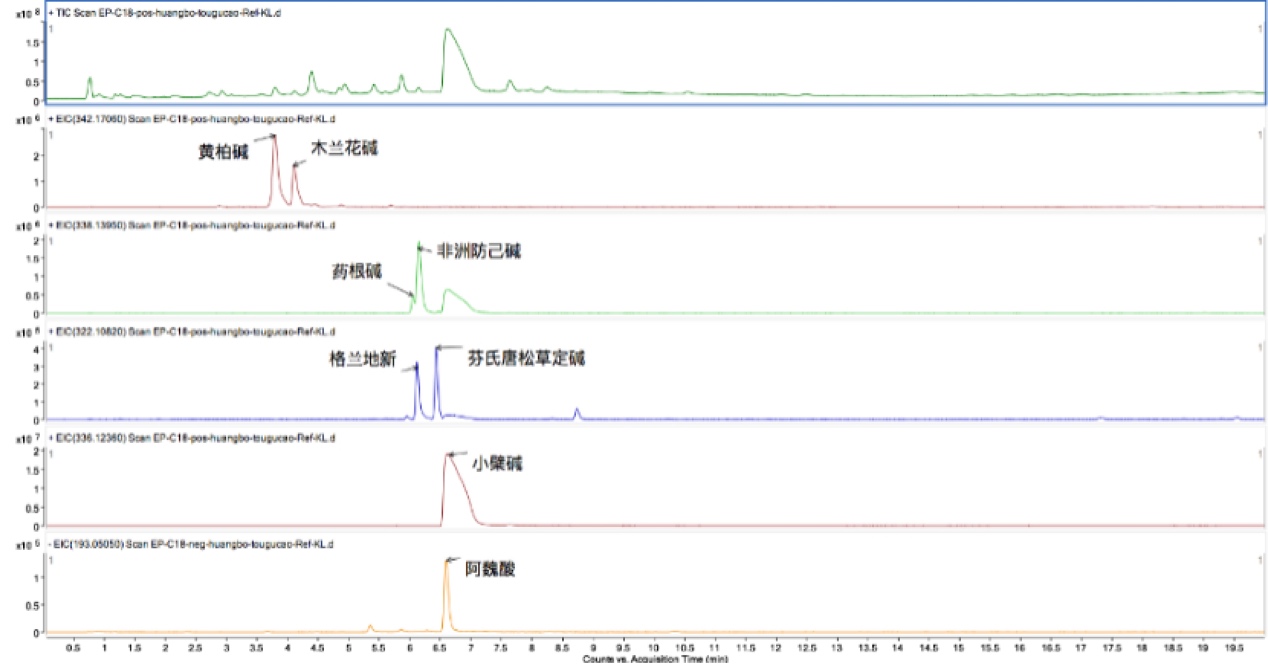


**Additional file 1: Fig. S1.** **The fingerprints of CQBP.** Peak number and identity, 1: phellodendrine; 2: magnoflorine; 3:jatrorrhizine; 4: tetrandrine; 5: columbamine; 6: phenanthrene herb and alkaloid; 7: berberine; 8: ferulic acid.
